# No evidence of handling‐induced mortality in Serengeti's African wild dog population

**DOI:** 10.1002/ece3.4798

**Published:** 2018-12-26

**Authors:** Craig R. Jackson, Emmanuel H. Masenga, Ernest E. Mjingo, Andrew B. Davies, Frode Fossøy, Robert D. Fyumagwa, Eivin Røskaft, Roel F. May

**Affiliations:** ^1^ Norwegian Institute for Nature Research (NINA) Trondheim Norway; ^2^ Tanzania Wildlife Research Institute (TAWIRI) Arusha Tanzania; ^3^ Department of Global Ecology Carnegie Institution for Science Stanford California; ^4^ Department of Biology Norwegian University of Science and Technology (NTNU) Trondheim Norway

**Keywords:** animal welfare, canid conservation, ethics, immobilization, radio telemetry

## Abstract

The disappearance of an endangered African wild dog population from Serengeti National Park (SNP) led to international debate centered around one question: were researchers to blame? The “Burrows' hypothesis” postulated that stress induced by research‐related immobilization and handling reactivated a latent rabies virus, eliminating the population. Insufficient data inhibited hypothesis testing, but since wild dogs persisted alongside SNP and have been studied since 2005, the hypothesis can be tested 25 years after its proposition. To be supported, wild dog immobilization interventions should have resulted in high mortality rates. However, 87.6% of 121 handled wild dogs (2006–2016) survived >12 months post‐handling. Some argued that viral reactivation would necessitate long‐term stress. Following immobilization, 67 animals were captured, transported, and held in a translocation enclosure. Despite the longer‐term stress, 95.5% survived >12 months. Furthermore, the stable number of wild dog packs in the ecosystem over the past decade, and lack of recolonization of SNP, strongly oppose Burrows' hypothesis. Instead, factors such as heightened levels of interspecific competition are likely to have contributed to the wild dog disappearance and subsequent avoidance of the Serengeti plains. Handling and radio telemetry are invaluable when studying elusive endangered species, yielding information pertinent to their conservation and management, and had no effect on Serengeti wild dog survival.

## 1 INTRODUCTION

Large carnivores have suffered significant global population declines, and protected areas are becoming increasingly important for their continued survival in the face of anthropogenic threats (Bauer et al., 2015; Riggio et al., 2013; Ripple et al., 2014). However, even within large protected areas carnivore populations can decline precipitously to the point of local extinction (Groom, Funston, & Mandisodza, 2014). Factors driving such events may not be immediately apparent, even when populations have been the subject of long‐term research and monitoring. An understanding of potential extinction‐causing factors, including the role of well‐intended human interventions, are vital to facilitate informed conservation and management actions within protected areas (Rosenblatt et al., 2014; Woodroffe & Ginsberg, 1998).

A case in point is that of the African wild dog (*Lycaon pictus*) population that formerly inhabited the grassland plains in Serengeti National Park (SNP), Tanzania. Studied since 1964, this population of endangered wild dogs declined and eventually disappeared entirely from the study area in 1991 (Burrows, Hofer, & East, 1994; Ginsberg, Mace, & Albon, 1995). The cause of the wild dogs’ decline and eventual demise was extensively debated among scientists at the time (Burrows et al., 1994; Burrows, Hofer, & East, 1995; Creel, Creel, & Monfort, 1997; Devilliers et al., 1995; East & Hofer, 1996; East, Hofer, & Burrows, 1997; Gascoyne et al., 1993; Ginsberg, Alexander, et al., 1995; Ginsberg, Mace, et al., 1995; Woodroffe, 2001). Yet more than 25 years later, consensus has not been reached. A controversial hypothesis implicated researchers and their handling of wild dogs as the driver of the extinction. Prior to their demise, serum samples showed that some of the population had been exposed to rabies and certain animals had significant rabies‐neutralizing antibody titers. This led Burrows (1992) to postulate that the stress associated with the immobilization and handling of wild dogs (in the absence of vaccination) for research purposes caused immunosuppression, resulting in the reactivation of a latent form of the rabies virus, thereby causing the death of the entire study population. Despite staunch opposition (see Methods), the hypothesis was vehemently defended by its proponents and the debate remains unresolved (Burrows, 2011; Burrows et al., 1994, 1995; East & Hofer, 1996; East et al., 1997).

The implications of this hypothesis extend beyond wild dogs in the Serengeti; not only was immobilization of wildlife periodically suspended in certain countries immediately thereafter, but also the notion of researcher‐induced extinction continues in the scientific literature (Crozier & Schulte‐Hostedde, 2014; Vander Wal, Garant, & Pelletier, 2014). More recently, Burrows (2011) attributed wild dog population declines in two southern African populations to researcher intervention, thereby perpetuating the validity of the original hypothesis. Furthermore, three handled packs in three different southern African protected areas succumbed to disease during 2016–2017; could this also have been due to researcher intervention? The hypothesis’ validity not only has ethical implications for research activities but is particularly important for the conservation and management of this and other endangered species. Therefore, resolving whether research intervention was to blame more than 25 years after the event is challenging, yet critically important.

**Figure 1:**
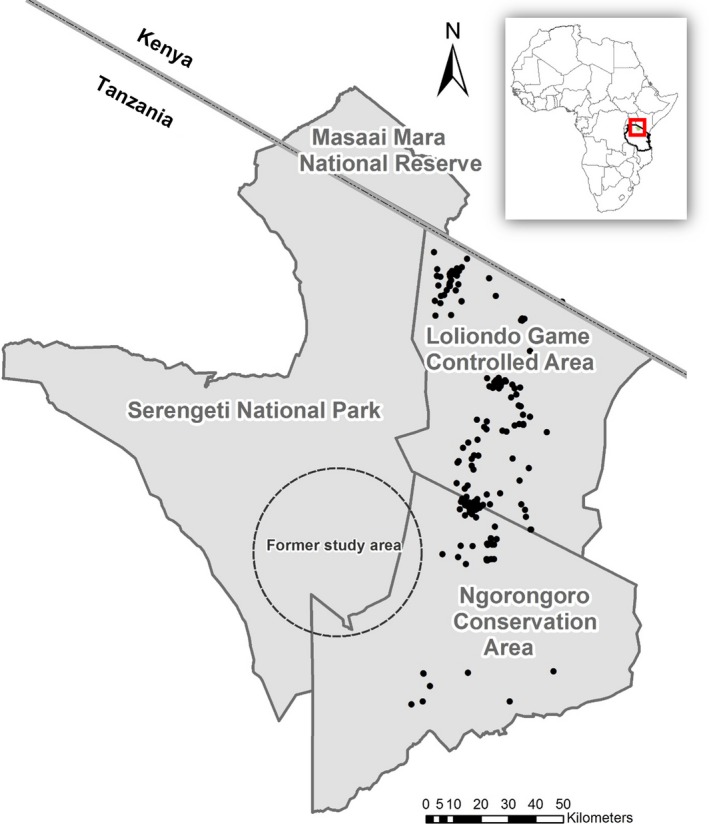
The Serengeti–Mara Ecosystem in northern Tanzania and southern Kenya. Black circles denote the location of resident packs observed during chance encounters or tracking of very high frequency (VHF) collared individuals between 2005 and 2009. The approximate location of the former study area is indicated by the large dashed circle

Although much of the scientific literature referred to the disappearance of the wild dogs from SNP as a population “extinction”, the population never went extinct within the broader region (Burrows et al., 1995; Lyamuya et al., 2016; Marsden, Wayne, & Mable, 2012). Packs persisted in the adjoining Loliondo Game Controlled Area (LGCA) and Ngorongoro Conservation Area (NCA) to the east of SNP (Figure 1) and numerous packs have occupied these areas for the past decade (Masenga, 2017). Sporadic sightings of transient groups as well as global positioning system (GPS) collar data (unpublished data) indicates that these packs occasionally enter SNP, thereby confirming accessibility and connectivity across the entire region, and that the resident wild dogs represent a single population (Marsden et al., 2012). The wild dog population thus survived in the eastern parts of the ecosystem and has been the subject of research and monitoring since 2005.

### 1.1 How to revisit and test the hypothesis 25 years later?

Low‐intensity monitoring of study animals and the collection of few biological samples prevented a conclusive, data‐derived consensus on the cause of the Serengeti wild dog disappearance. Researchers found no evidence of handling‐induced mortality in other ecosystems (Ginsberg, Alexander, et al., 1995), yet the proponents of Burrows’ hypothesis discredited such studies, stating that “If intervention caused immunosuppression and increased disease‐mediated mortality among adult pack members, then logically any test of this idea should be conducted on data from ecosystems where (a) wild dogs contact pathogens that are lethal to adults; (b) rates of exposure to lethal pathogens are similar to that for rabies in the Serengeti; (c) types and levels of interventions sustained by packs are similar to those applied in the Serengeti” (East et al., 1997). Using data from wild dogs in the same ecosystem would therefore present an ideal study opportunity to test the hypothesis.

Since wild dogs did survive in the Serengeti ecosystem and have been the subject of an active research program that included immobilization and radio collaring, we aimed to test Burrows’ hypothesis while satisfying the above‐mentioned criteria. Firstly, rabies and canine distemper virus are prevalent in the ecosystem, including LGCA and NCA (Cleaveland & Dye, 1995; Cleaveland et al., 2007; Gascoyne et al., 1993; Goller et al., 2010; Lembo et al., 2008; Roelke‐Parker, Munson, Packer, & Kock, 1996), and are lethal to adult wild dogs (Goller et al., 2010; Hofmeyr, Bingham, Lane, Ide, & Nel, 2000). During a five‐year period alone (2002–2007), 128 cases of rabid domestic dogs were reported in LGCA and NCA (Lembo et al., 2008). Secondly, contact with domestic dogs increases exposure to rabies (Woodroffe et al., 2012) and therefore, rates of exposure are expected to be at least the same or higher than those experienced 20 to 30 years ago in SNP. This is because (a) the domestic dog population, a reservoir host for rabies (Lembo et al., 2008), has increased in size (Craft et al., 2017), with an annual growth rate of up to 8% in certain parts of the ecosystem (Czupryna et al., 2016) and (b) the surviving wild dog population occurs sympatrically with domestic dogs, and it is thus reasonable to assume that the current wild dog population has even greater rates of exposure to the rabies virus than the portion of the wild dog population formally resident within SNP, which is almost entirely free of domestic dogs. Several cases of rabies have been reported from the areas adjoining SNP post‐1991 (Lembo et al., 2008), and over 40% of village members in NCA reported the presence of wild dogs at their households (Czupryna et al., 2016), confirming the sympatric occurrence of domestic dogs and wild dogs. Thirdly, wild dogs are exposed to similar types of interventions, that is immobilization and collaring (Masenga et al., 2016), and the same types of handling that allegedly resulted in individual and pack mortality in SNP (Burrows et al., 1994).

To ensure that the underlying mechanistic basis of Burrows’ hypothesis was met, we used data from the same Serengeti wild dog population and assessed whether handling led to any detectable increases in mortality. Between 2006 and 2016, 121 wild dogs were immobilized and handled within the Serengeti Ecosystem, 45 of which were radio‐collared. Burrows et al. (1994) reported that “handled individuals were significantly less likely to survive for 12 months after the date of first handling”. We therefore assessed the survival of the handled wild dogs for 12 months post‐handling in an attempt to determine whether increased mortality was evident following short‐term and longer‐term handling interventions. Additionally, we assessed whether there had been any recolonization of wild dogs in SNP because if researcher intervention alone was responsible for the disappearance of wild dogs on the Serengeti plains, then recolonization over the past 25 years would have been probable given the persistence of numerous wild dog packs immediately alongside the former study area and the cessation of wild dog research within SNP.

## 2 MATERIALS AND METHODS

### 2.1 Study area and wild dog populations

The Serengeti–Mara Ecosystem (Figure 1) encompasses over 30,000 km^2^ of wildlife‐dominated land in northern Tanzania and south‐western Kenya. The well‐protected Serengeti National Park (SNP; 14 763 km^2^) and Maasai Mara National Reserve (MMNR; 1,510 km^2^) form the heart of the system and are bordered by areas of differing land use. The two largest adjoining areas with multiple forms of land use are Loliondo Game Controlled Area (LGCA; 4,300 km^2^) and Ngorongoro Conservation Area (NCA; 8,300 km^2^). Land use in LGCA is diverse and includes several human settlements and villages, dominated by the livestock‐herding Maasai tribe, nature‐based tourism, trophy hunting concessions, and subsistence agriculture. Ngorongoro Conservation Area borders both LGCA and SNP and in addition to wildlife is home to sizeable Maasai and livestock populations. When the Serengeti wild dog study commenced in 1964, a 3,000 km^2 ^study area dominated by grassland plains was selected (Frame, Malcolm, Frame, & Lawick, 1979). In 1973, the study area was expanded northwards to include a total of 5,200 km^2 ^and comprised 4,200 km^2^ of short and medium grassland and 1,000 km^2^ of surrounding open *Acacia* woodlands (Frame et al., 1979).

Following their disappearance from the Serengeti plains, the wild dog population survived in LGCA and NCA. Locals in LGCA and NCA saw wild dogs regularly for several decades, both before and after their disappearance from the Serengeti plains (Lyamuya et al., 2016). Genetic evidence further indicates that these wild dogs are genetically similar to the wild dogs formally resident on the Serengeti plains, ruling out recolonization from elsewhere (Marsden et al., 2012). In combination, this evidence confirms that the Serengeti wild dog population did not go extinct, but survived in the eastern part of the ecosystem, with the population currently comprised of 120 animals in ten packs (Masenga, 2017). The Tanzania Wildlife Research Institute (TAWIRI) has monitored this wild dog population since 2005, including the use of radio collars, allowing us to test specific predictions related to Burrows’ hypothesis.

### 2.2 A timeline of wild dog research and recorded mortality

**Figure 2:**
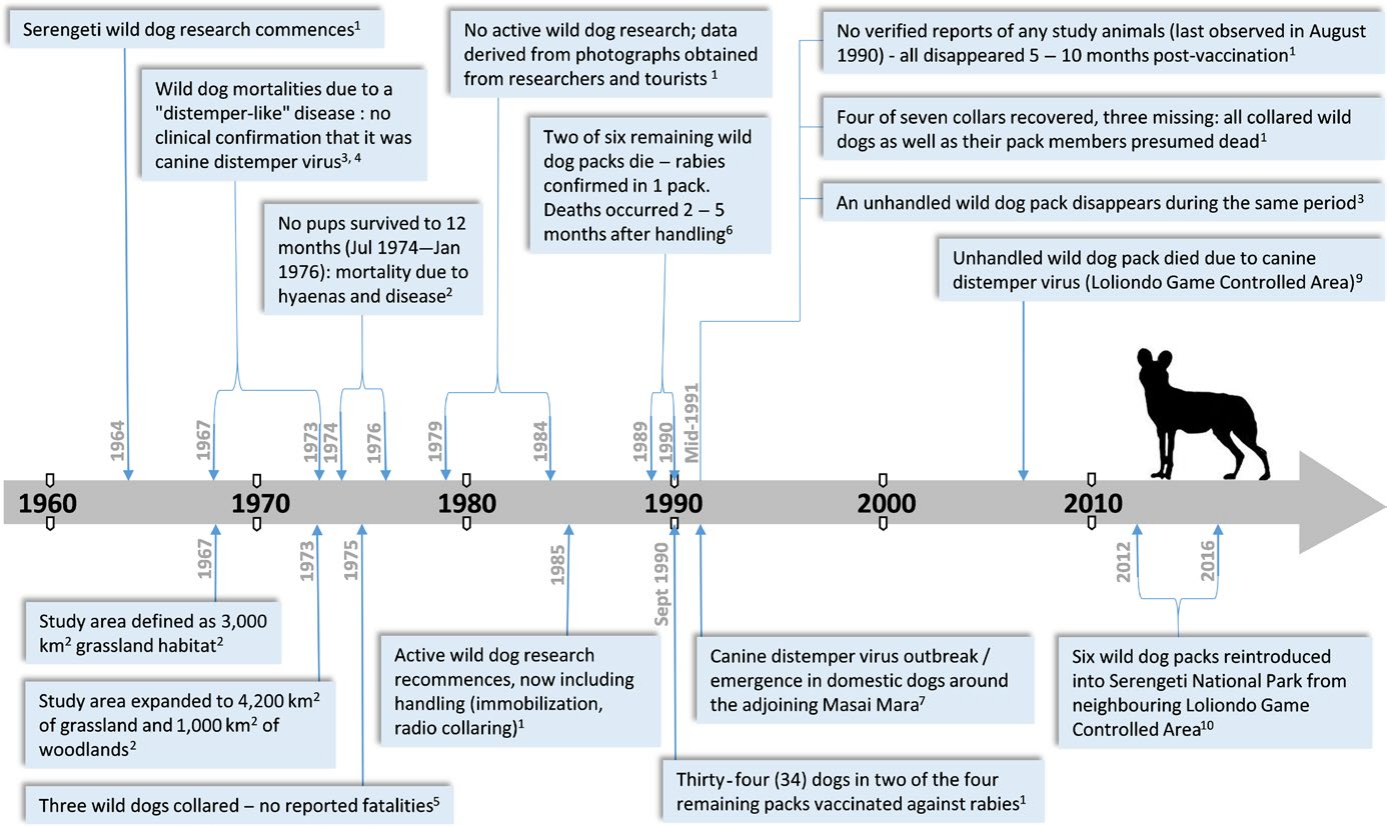
A timeline highlighting dates of events or observations relevant to understanding and assessing the potential causes of the localized disappearance of the Serengeti wild dog population. Sources: 1. Burrows et al. (1994), 2. Frame et al. (1979), 3. Schaller (1972), 4. Malcolm (1979), 5. Frame and Frame (1981), 6. Dye (1996), 7. Alexander and Appel (1994), 8. Gascoyne and Laurenson (1994), 9. Goller et al. (2010), 10. Masenga (2017)

A timeline of published records on wild dog handling, mortality, and other events relevant to the populations’ persistence in the Serengeti Ecosystem are presented in Figure 2. In 1970, the Serengeti study population numbered an estimated 95 wild dogs in 12 packs, the vast majority of which occurred on the open grassland plains in SNP (Burrows et al., 1994; Ginsberg, Mace, et al., 1995). Little research was conducted during the early 1980s, but work recommenced in 1985 and saw researchers immobilize and collar wild dog packs. Between 1985 and 1990, a total of five Serengeti packs died or disappeared (Burrows, 2011), and rabies was confirmed in one instance (Gascoyne & Laurenson, 1994). These deaths occurred two to five months after handling by researchers for radio collar deployment. In an attempt to protect the remaining packs, a vaccination program was initiated in 1990. Despite this, the remaining seven packs died five to 12 months after vaccination and the wild dog population was declared locally extinct (Burrows, 1992; Burrows et al., 1994). Although Burrows et al. (1994) maintained that the final demise was due to rabies, no samples were collected in the Serengeti, and there is no evidence that rabies caused the post‐vaccination wild dog deaths (Gascoyne & Laurenson, 1994). Instead, others suggested that it might have been canine distemper virus, which had emerged in the ecosystem at a similar time (Alexander & Appel, 1994). If true, this would also account for why rabies vaccinations failed to protect the remaining wild dogs against the disease outbreak.

Stress, and the potential inhibitory effect that elevated glucocorticoid levels may have on the immune system, are central to Burrows’ hypothesis. No data on glucocorticoids levels, or fluctuations therein following handling, are available for the Serengeti wild dogs. Stress per se is not necessarily harmful; short‐term stress responses are adaptive and may be beneficial to an organism's survival, while a prolonged stress response may be harmful (Creel et al., 1997). While handling does increase wild dog stress levels in the short‐term, there is no evidence that this is persistent enough to result in disease reactivation (Devilliers et al., 1995).

### 2.3 Testing Burrows’ hypothesis

#### 2.3.1 Prediction 1: Effects of handling‐induced stress on survival

The handling referred to by Burrows was all of a relatively short duration, yet deemed sufficient to evoke the reactivation of latent rabies (Burrows et al., 1995). Consequently, for Burrows’ hypothesis to be supported, the presence and potential exposure to rabies (as well as canine distemper virus) should see the handling of wild dogs in the Serengeti Ecosystem result in high rates of mortality following stress‐induced viral reactivation. To assess this prediction, we collated all available data on wild dog immobilizations conducted within the Serengeti Ecosystem post‐1991. All immobilizations during this period were carried out by TAWIRI veterinarians, and drugs were administered via darting rifle; the same technique utilized prior to 1991. A total of 121 wild dogs were immobilized between June 2006 and July 2016, 45 of which were radio‐collared. We assessed individual survival to 3, 6, and 12 months post‐immobilization.

#### 2.3.2 Prediction 2: Effects of short‐term and long‐term stress on survival

Opponents to Burrows’ hypothesis argued that short‐term stress from relatively brief immobilizations would be insufficient to reactivate latent viruses, and that longer‐term (chronic) stress would be required for this to potentially occur (Devilliers et al., 1995). Between 2012 and 2016, six wild dog packs were immobilized and captured in LGCA, and thereafter relocated to SNP (Masenga, 2017). The comparatively long‐term handling that included immobilization, physical handling while loading into crates, relocation by road, and prolonged confinement in enclosures prior to release into an unfamiliar area, would be likely to result in far greater and prolonged stress levels than comparatively brief immobilization and radio collaring. Consequently, in accordance with Burrows’ hypothesis, high rates of mortality would be predicted for these animals. To contrast the potential effects of short‐ versus long‐term stress (handling), we assessed survival to 12 months post‐handling of wild dogs that were handled and relocated (*n* = 67) with those that were not relocated but released after a short handling period (*n* = 54).

#### 2.3.3 Prediction 3: Recolonization

For the past decade, several wild dog packs have occupied the area immediately alongside the Serengeti plains where wild dogs were studied between 1964 and 1991 (Masenga, 2017). Following the wild dogs’ disappearance from SNP, four to six packs persisted in LGCA and NCA (Burrows et al., 1994). The number of packs more than doubled in subsequent years, with 13 packs recorded in LGCA alone during 2012 (Masenga, 2017). Wild dog populations have the ability to rapidly increase in size (Woodroffe, 2011), and dispersal and pack formation have been recorded in the population (Masenga et al., 2016). New packs need to establish territories (Fuller et al., 1992) and the lack of wild dogs on the Serengeti plains post‐1991 would have provided the necessary territorial voids immediately alongside the other resident packs (Jackson, Groom, Jordan, & McNutt, 2017; Mcnutt, 1996). Accordingly, we predict that if handling alone was responsible for the extinction (Burrows et al., 1994), the former study area would have been recolonized by the packs and dispersing groups occurring and originating immediately alongside because the reason for the demise of the SNP packs (researcher intervention) had been eliminated. Furthermore, six packs were reintroduced into SNP between 2012 and 2016, further increasing the probability of recolonization. Successful recolonization would support Burrows et al. (1994)s argument that ecological factors, such as interspecific competition, did not play a significant role in the study population's demise. In contrast, a lack of recolonization over a 25‐year period would provide evidence against the hypothesis that researcher intervention alone was responsible for the decline and disappearance of the Serengeti wild dogs. Researchers have a permanent presence in and around the formerly inhabited area which, in addition to the large number of tourists in this area, would have detected the reestablishment of wild dogs. We additionally used data from an extensive camera‐trap survey, which covered 1,125 km^2^ of the area (Swanson et al., 2015), to evaluate whether wild dogs were detected and therefore potentially recolonized the area.

## 3 RESULTS

### 3.1 Prediction 1: Effects of handling‐induced stress on survival

Of the 121 animals immobilized between 2006 and 2016, 87.6% (*n* = 106) survived at least 12 months post‐handling, while 91.7% (*n* = 111) and 95.9% (*n* = 116) survived more than 6 and 3 months post‐immobilization, respectively. The high survival rate does not support the hypothesis that handling negatively impacts wild dog survival.

### 3.2 Prediction 2: Effects of short‐term and long‐term stress on survival

Between 2012 and 2016, 67 wild dogs from six different packs were captured and translocated to SNP, after a mean period of 313 days in enclosures (range: 76 to 499 days). Survival of these individuals was high: 95.5% (*n* = 64 of 67) of wild dogs survived more than 12 months post‐handling. Furthermore, all six translocated packs survived more than 12 months post‐handling. In contrast, survival to 12 months post‐handling for wild dogs that were immobilized, but not captured and translocated, was 77.8% (*n* = 42 of 54). Thus, despite the longer‐term handling and stress resulting from the capture and translocation process, mortality was higher in non‐translocated packs that were exposed to short‐term handling and stress, indicating that factors other than handling had a greater effect on survival. Therefore, longer‐term stressful interventions did not evoke disease outbreaks, and the high survival rate does not support Burrows’ hypothesis.

### 3.3 Prediction 3: Recolonization

Monitoring of wild dogs in the ecosystem has continued since 2005. Over the past decade, the population has increased, with as many as 13 known packs within the immediate area at times (Masenga, 2017). Although these packs were in close proximity to SNP and the former study area, no recolonization of the Serengeti plains has occurred. This area is frequently visited by tourists and the TAWIRI researchers have a permanent presence within the park. Consequently, recolonization by these conspicuous carnivores would almost certainly have been rapidly detected. The wild dog's failure to recolonize is also evident from a large‐scale camera trapping survey (“Snapshot Serengeti”) that covered 1,125 km^2^ of the former study area. Between 2010 and 2013, a total of 225 cameras accumulated 1.2 million images over 99,241 camera‐trap days (Swanson et al., 2015). This large number of images resulted in 334,671 species‐specific capture events, documenting the presence of 40 mammalian species, but not wild dogs (table 1 in Swanson et al., 2015). In comparison, these capture events included 1,272 images of cheetah (*Acinonyx jubatus*), another carnivore that occurs at a low density, as well as 4,266 of lion (*Panthera leo*), and 5,303 of spotted hyaena (*Crocuta crocuta*) (Swanson et al., 2015).

Dispersal and pack formation did occur outside SNP with packs establishing both within the LGCA and NCA as well as further away in Kenya (Masenga et al., 2016). However, no new or established packs returned to occupy the Serengeti plains region where they were studied between 1964 and 1991. Transient dispersal groups were recorded within the former study area soon after the disappearance and sporadically in the years thereafter (Burrows et al., 1995), confirming the area's accessibility to wild dogs. Recolonization did not occur despite cessation of wild dog research (that Burrows’ hypothesis attributed as causal for the extinction) in the area. Moreover, there was adequate prey availability (Ginsberg, Mace, et al., 1995), and a territorial void would have been created by the lack of resident wild dog packs. The wild dogs’ failure to recolonize SNP prompted the initiation of the wild dog reintroduction program in 2012 (Masenga, 2017). Despite being released in SNP and within relatively close proximity to the plains, none of the six reintroduced wild dog packs occupied the previously inhabited habitat (Masenga, 2017), further highlighting the wild dog's avoidance thereof. Within the first 12 months, three of the reintroduced packs had established territories outside SNP, two packs along the north‐western boundary and only one entirely inside SNP, in rugged terrain to the west of the former (plains) study area (Masenga, 2017).

## 4 DISCUSSION

Burrows’ hypothesis postulates that the decline and disappearance of the African wild dog population on the Serengeti plains was a direct result of researcher‐induced disease outbreaks. Despite the resistance of the scientific community at large to accept this hypothesis, the proponents thereof have consistently defended it (Burrows, 2011; Burrows et al., 1994, 1995; East & Hofer, 1996; East et al., 1997). Using a multifaceted approach and data from the same wild dog population, where disease is still prevalent, we found no support for Burrows’ hypothesis. Survival following both short‐ and long‐term human interventions was high and no decline in the number of resident packs occurred (Masenga, 2017). Furthermore, recolonization of the former study area has not occurred during the past 25 years, despite active reintroduction attempts and the cessation of wild dog research, strongly suggesting that researcher intervention alone was not responsible for the wild dog decline and eventual disappearance from SNP in 1991. Our results are supported by earlier work showing that the survival of adult and yearling wild dogs in reintroduced packs (all individuals captured and handled) and free‐ranging packs (majority of individuals unhandled; maximum of two adults per pack handled for radio collar deployment) did not differ (Masenga, 2017).

Both rabies and canine distemper virus can be fatal to wild dog individuals and packs (Goller et al., 2010; Hofmeyr et al., 2000; Kat, Alexander, Smith, & Munson, 1995) and both diseases were present within the Serengeti Ecosystem at the time of the wild dogs’ disappearance (Alexander & Appel, 1994; Cleaveland & Dye, 1995; Gascoyne et al., 1993). It is likely that one of these diseases caused the final disappearance of the study packs during 1990–1991, but the lack of biological samples prevented the determination thereof. Burrows’ hypothesis was entirely focused on rabies, yet canine distemper virus was another potential cause of mortality and a disease to which the wild dogs had not been vaccinated (Ginsberg, Mace, et al., 1995; Macdonald, 1992). Indeed, less than three years later, canine distemper virus was responsible for the death of a third of SNP's lion population (Roelke‐Parker et al., 1996).

More concerning, however, was the assumption that all wild dog individuals and packs had died. Little data on the actual number of dead wild dogs and packs were presented, and a large number of “deaths” were entirely circumstantial. The two Serengeti packs (*n* = 34 individuals), that were vaccinated in September 1990, had disappeared five to ten months after vaccination (Burrows et al., 1994). Four of seven radio collars were recovered, while three were unaccounted for (Burrows et al., 1994). Instead of verified mortality data, Burrows et al. (1994) assumed that collared and uncollared wild dogs had the same mortality rate, and by means of extrapolation reasoned that at least 57% (4 of 7) of all wild dogs had died. These authors proceeded even further and suggested that the entire population had died based on a lack of resightings. While four individuals may have died, due to unknown reasons, this cannot be used as evidence for the death of 34 individuals. This is particularly pertinent given that during the five months post‐vaccination, the two wild dog packs spilt up and formed five packs (Burrows et al., 1994). Such dynamics have been observed in the Serengeti Ecosystem to result in dispersing pack members covering hundreds of kilometers (Masenga et al., 2016). Lacking satellite GPS telemetry, such movements would most likely have gone undetected, and the study animals would not have been sighted again. Failure to observe individuals or packs in their former range can therefore not be equated with pack mortality.

Moreover, pack fission and new pack formation occurred during a period when emigration from and immigration to the study area was confirmed (Burrows et al., 1994), increasing the probability of disease transmission between distant parts of the ecosystem. Post‐dispersal animals that had immigrated into packs survived significantly shorter than pre‐dispersers (Burrows et al., 1994). While it was argued that post‐dispersal individuals were exposed to increased social stress in their new packs, making them more susceptible to stress‐induced disease reactivation (Burrows et al., 1994), it is perhaps more likely that these individuals had been exposed to disease during their dispersal movements.

Previous studies found no evidence of handling‐induced mortality using data from wild dog populations in five different ecosystems (Ginsberg, Alexander, et al., 1995). However, Burrows et al. (1995) argued that this finding was not relevant to the Serengeti wild dog extinction because the prevalence of and wild dog exposure to pathogens in those ecosystems was unknown. Instead, these authors argued that the potential absence of disease would prevent wild dog exposure and thus the development of a latent virus from researcher‐induced stress. By working in the same ecosystem, with the same wild dog population that is still vulnerable to disease, we circumvented this problem, yet found no evidence of researcher‐induced mortality in the Serengeti region. Ironically, Burrows (2011) invoked the handling‐induced mortality hypothesis to explain declines in the Moremi Game Reserve (Botswana) and Kruger National Park (South Africa) wild dog populations, despite specifically mentioning these populations previously when refuting Ginsberg, Alexander, et al. (1995). Such broad‐scale application of the handling‐induced mortality hypothesis without clear evidence highlights the necessity of determining the true cause of the Serengeti wild dog disappearance before unnecessary restrictions on wild dog, and possibly other endangered species, research is implemented elsewhere.

We found that wild dogs that were immobilized, captured, transported, and held for several months (thereby exposed to prolonged stress) survived longer than those that were only briefly immobilized and thereafter released. This opposes the idea of fatal stress‐induced reactivation of rabies. Furthermore, at least three wild dogs were collared in SNP in 1975, yet no deaths or disease outbreaks were reported (Frame & Frame, 1981), whereas an unhandled wild dog pack of seven individuals died during the extinction period (Gascoyne & Laurenson, 1994). Therefore, Burrows’ hypothesis does not hold true in the Serengeti ecosystem before, during, or after the disappearance of the Serengeti wild dogs.

According to the data presented by Burrows et al. (1994, 1995), no other factors could explain the demise of the Serengeti wild dog population. Consequently, the recovery and expansion of the wild dog population in the eastern parts of the ecosystem should have resulted in recolonization. Despite this population having been monitored since 2005, no packs have taken up residence on the Serengeti plains. Failure to recolonize suggests that other factors are preventing the wild dog's return to the plains. These same factors may have contributed to the population decline preceding and leading to the eventual disappearance. The majority of arguments in response to Burrows’ hypothesis focused on the controversial hypothesis itself, which only addressed the final stage of the wild dogs’ demise, yet “the question about why they had long been in decline is arguably the more important” (Dye, 1996). More than two decades later, the wild dog's failure to recolonize the Serengeti plains, despite their occurrence immediately alongside, may be equally important, as well as revealing, as to the cause of their disappearance.

What then could have caused the wild dog population decline and disappearance, and still hinder recovery decades later? Evidence from several ecosystems indicates that wild dogs are vulnerable to competition from lions and hyaenas, both through direct mortality (Groom, Lannas, & Jackson, 2017; Woodroffe & Ginsberg, 1999) and kleptoparasitism (Carbone et al., 2005). The high risk posed by lions results in wild dogs avoiding them at all times (Webster, McNutt, & McComb, 2012) and, at the landscape scale, wild dog densities are inversely correlated with lion and spotted hyaena densities (Mills & Gorman, 1997). During the period of the Serengeti wild dog population decline, the spotted hyaena population increased by 150% (from 2,200 to 5,500) and similarly large increases were recorded in the lion population (Burrows et al., 1994), with concomitant decreases in wild dog pup survival and adult longevity (Ginsberg, Mace, et al., 1995). During the 1960s and 1970s, fewer than half of Serengeti wild dog kills were attended by hyaenas, increasing to 85% during the 1980s, and likely preventing wild dogs meeting their energy requirements (Carbone et al., 2005). Such effects were already apparent during the 1970s, when competition with hyaenas for food and disease resulted in no wild dog pups surviving to 12 months of age between July 1974 and January 1976 (Frame et al., 1979).

The wild dog population persisted in the eastern part of the ecosystem and mostly in LGCA (Masenga, 2017). LGCA is comprised of heterogeneous savanna habitat that differs largely from the homogenous, flat Serengeti grasslands from which the wild dogs disappeared. Heterogeneous habitats provide variability in resource abundance and distribution, affecting species interactions (Gorini et al., 2012). Lions and hyaenas respond to this resource variability and, as the dominant apex carnivores, select the most resource‐rich habitats, avoiding rugged areas, for instance, in favor of prey‐rich flatter terrain (Mills & Gorman, 1997). The resulting spatial variability in lion and hyaena densities facilitates avoidance by subordinate wild dogs (Mills & Gorman, 1997) and the development of a landscape of fear where high‐risk habitats are avoided and competition refuges are actively selected, particularly during the vulnerable denning season when rugged and densely vegetated areas, with fewer lions, are selected by wild dogs (Davies, Marneweck, Druce, & Asner, 2016; Jackson et al., 2014). Indeed, fluctuations in lion densities have been shown to elicit changes in wild dog behavior and habitat selection (Groom et al., 2017). In the absence of strong top‐down regulation, behavioral flexibility would likely see profitable habitats, such as the plains, being exploited. Lower interspecific competition thereby likely played a significant role in the wild dog population's persistence on the Serengeti plains prior to 1991 (Swanson et al., 2014), while higher densities of lions and hyaenas today inhibits recolonization of these habitats (Ginsberg, Mace, et al., 1995). We postulate that the disappearance from the Serengeti plains was instead merely a range contraction driven by increasing competitor densities with an outbreak of disease dealing the final blow to the remaining individuals and had little to do with researcher‐induced mortality.

Many threatened species are difficult to study due to their cryptic habits and/or low densities (Creel, 1996). Advocates of Burrows’ hypothesis argue that, due to the supposed negative effects, research and monitoring of such species should be conducted using entirely non‐invasive techniques. While animal welfare and ethical considerations should remain paramount, much of the information pertinent to management and conservation of threatened species would be impossible to attain in the absence of radio telemetry and other techniques that require researcher intervention (Creel, 1996). Conserving wide‐ranging carnivores is particularly challenging in an increasingly human‐dominated world, and the probability of conservation initiatives being successful are greatly increased by a thorough understanding of the nuances of species’ behavior and ecology. Consequently, in the case of the African wild dog, information gained through research involving radio telemetry and other interventions has most likely contributed to the conservation of the species as a whole, rather than compromised it.

## CONFLICT OF INTERESTS

We have no competing interests.

## AUTHOR CONTRIBUTIONS

CRJ conceived the study, analyzed the data, and drafted the manuscript. EHM, EEM, RDF conducted wild dog immobilization, collected wild dog survival data, and contributed to the writing of the manuscript. ABD, FF, ER, and RFM contributed to the study design and helped draft the manuscript. All authors read and approved the manuscript for publication.

## Data Availability

The dataset will uploaded as supplementary material upon acceptance of the manuscript.
